# Acute aortic dissection masquerading as large bowel obstruction: a case report

**DOI:** 10.1186/s43044-026-00775-y

**Published:** 2026-09-17

**Authors:** Olarinde Jeffrey Oluwademilade Nee Ogunmola, Azemobor Osigbeme, Henry Omagbeitse Abiyere, Eunice Oluremi Olabinri, Tolulope Fasanmi Kolawole

**Affiliations:** 1 Division of Cardiology, Department of Medicine, Federal Teaching Hospital Ido-Ekiti, Ekiti State, Nigeria; 2 Department of Medicine, Afe Babalola University, Ado-Ekiti, Nigeria; 3 Department of General Surgery, Federal Teaching Hospital Ido-Ekiti, Ekiti State, Nigeria; 4 Department of Radiology, Federal Teaching Hospital Ido-Ekiti, Ekiti State, Nigeria; 5 Division of Pulmonology, Department of Medicine, Federal Teaching Hospital Ido-Ekiti, Ekiti State, Nigeria

**Keywords:** Aortic dissection, Abdomen, Surgery, Hypertension, Case report

## Abstract

**Background:**

Acute aortic dissection (AD) is a life-threatening disorder. A significant diagnostic pitfall is its ability to mimic an acute surgical abdomen, particularly in Stanford type B dissections. The aim of this report is to underscore the lifesaving step of appropriate imaging to probe the vascular integrity of the aorta before surgical intervention in a hypertensive patient that presented with classic features of large bowel acute intestinal obstruction.

**Case presentation:**

A 50-year-old hypertensive male presented with a one-week history of constipation, abdominal distension, and colicky pain, initially diagnosed as large bowel obstruction. Cardiologist review was requested due to persistent severe hypertension. Further evaluation with a contrast-enhanced CT imaging revealed an extensive Stanford type B (DeBakey type IIIb) aortic dissection involving the arch and abdominal aorta. The patient was managed conservatively with parenteral labetalol and opioid analgesics before transfer to a facility of his choice.

**Conclusion:**

In the setting of acute abdomen and severe hypertension, imaging to probe the vascular integrity of the aorta is essential to avoid life-threatening, inappropriate surgical interventions. The primary “take-away” lesson is the need for high suspicion of AD in hypertensive patients with abdominal symptoms.

**Supplementary Information:**

The online version contains supplementary material available at https://doi.org/10.1186/s43044-026-00775-y.

## Introduction

Aortic dissection (AD) is the most common catastrophic event affecting the aorta [1]. While sudden chest pain is the classic symptom, approximately 38% of cases are missed during initial evaluation due to diverse clinical manifestations, and 5% to 15% involve gastrointestinal symptoms [2–5]. Recent medical reports indicate that the global incidence of acute aortic dissection (AAD) typically ranges between 3 and 16.9 cases per 100,000 individuals per year [6]. A comprehensive systematic review of population-based studies established a pooled global incidence of 4.8 per 100,000 individuals per year [7].

There are no validated clinical decision rules to help identify AAD despite it requiring urgent recognition [1, 2]. The current case is unique because the patient presented with symptoms solely focused on gastrointestinal issues, masquerading as a large bowel obstruction.

## Patient information

A 50-year-old Nigerian male was referred to the Federal Teaching Hospital Ido-Ekiti (FETHI). His primary concerns were constipation, progressive abdominal swelling, and colicky abdominal pain over one week. He had a 20-year history of hypertension and bladder outlet obstruction secondary to benign prostatic hypertrophy. He was a non-smoker and not diabetic.

## Clinical findings

On physical examination, the patient was in painful distress with tachycardia (120 bpm) and a blood pressure of 180/130 mmHg. The abdominal examination showed distension, tenderness, hyperactive bowel sounds, and scanty hard faecal matter on rectal examination. Cardiologist review noted a blood pressure of 200/140 mmHg, an apex beat in the fifth left intercostal space lateral to the midclavicular line, and a loud aortic component of the second heart sound.

### Timeline

**Table:** 

Timeframe	Clinical Event & Findings	Action / Intervention
Day 1–6	Onset of constipation, progressive abdominal swelling, and colicky pain.	Plain abdominal X-ray, patient monitored symptoms at home and peripheral health centre.
Day 7	Assessment at peripheral centre: large bowel acute intestinal obstruction.	Soap and water enemas (x3); referral to tertiary centre (FETHI).
Day 8 (Arrival)	Presentation at AED; Pulse: 120 bpm, BP: 180/130 mmHg.	General Surgery team initiated conservative management for acute partial intestinal obstruction.
Day 8 (Review)	Persistent pain, headache, and back pain; BP rose to 200/140 mmHg.	Cardiologist review requested; “red flags” for vascular etiology identified.
Day 8 (Imaging)	Emergent contrast-enhanced CT scan.	Diagnosis: Stanford Type B/DeBakey Type IIIb Aortic Dissection.

### Diagnostic assessment

**Fig. 1:**
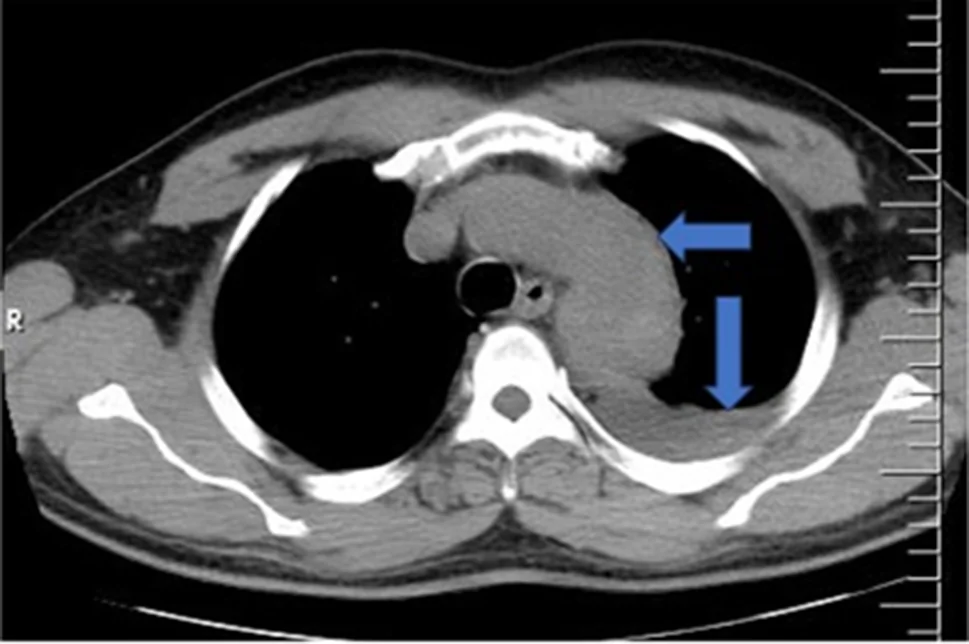
Pre-contrast axial chest CT image at the level of the aortic arch demonstrating significant aneurysmal dilation (horizontal arrow) and a left-sided pleural effusion (arrow pointing downward), often a sign of periaortic inflammation or incipient rupture in the setting of AAD

**Fig. 2:**
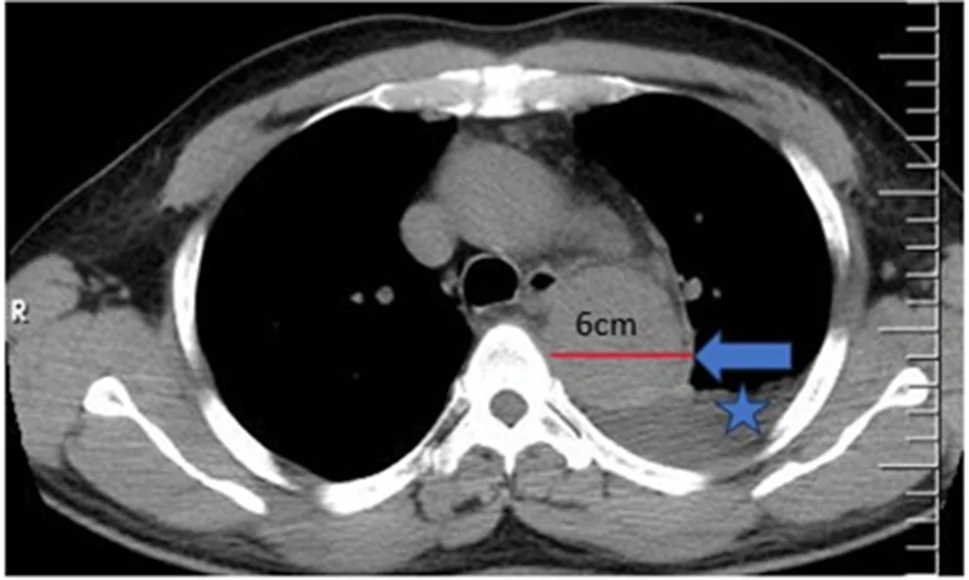
Pre-contrast chest CT showing the descending thoracic aorta at its widest point (arrow), measuring approximately 6 cm in diameter, and a left-sided pleural effusion (star)

**Fig. 3:**
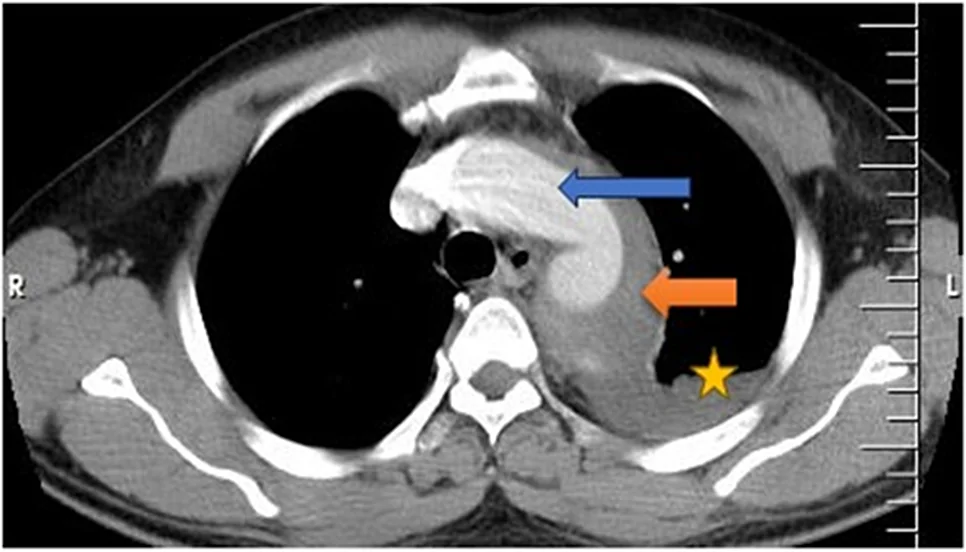
Post-contrast axial chest CT image with significant aortic aneurysm showing the dissected aortic arch. True lumen (blue arrow), false lumen (orange arrow), and a left-sided pleural effusion (star), often a sign of periaortic inflammation or incipient rupture

**Fig. 4:**
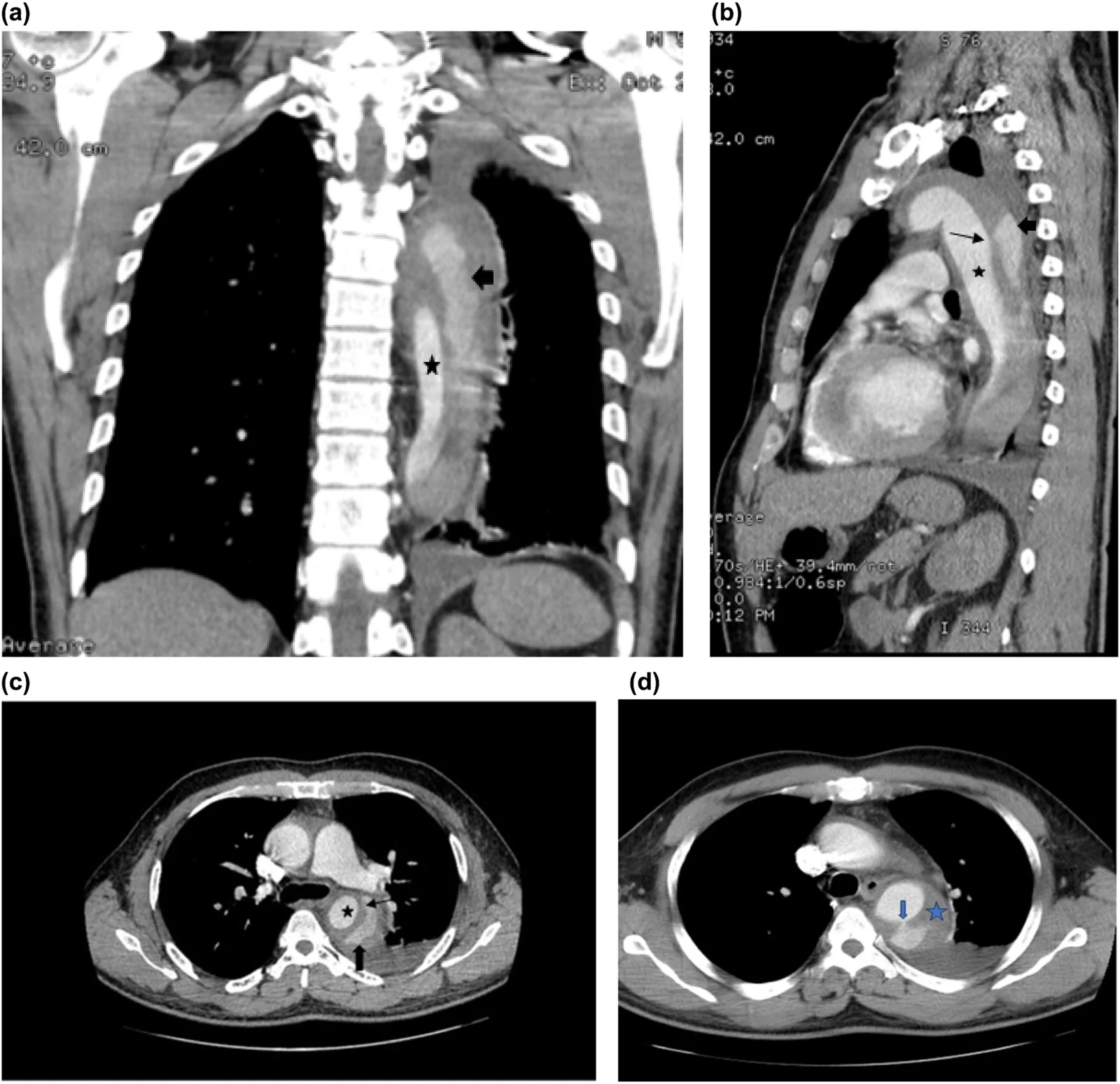
**a**: Post-contrast high-resolution coronal plane of chest CT scan revealing aortic dissection extending from the arch (after the left subclavian artery) to the descending thoracic aorta showing the true lumen (star) and false lumen (big arrow) and the presence of adjoining mural thrombi. **b**: Post-contrast sagittal chest CT scan of the aortic arch and descending thoracic aorta revealing an intimal flap (small arrow) separating the true (star) and false lumens (big arrow) and the presence of adjoining mural thrombi. **c**: Post-contrast axial chest CT scan of the descending thoracic aorta revealing an intimal flap (small arrow) separating the true (star) and false lumens (big arrow), and the presence of adjoining mural thrombi. **d**: Post-contrast axial chest CT of the descending thoracic aorta highlighting the communication ‘entry tear’ (arrow) between the true and false lumens and the presence of adjoining mural thrombi (star)

The initial diagnosis was acute partial intestinal obstruction. Cardiologist review was requested due to persistent severe hypertension. The persistence of severe hypertension and the ‘loud’ aortic component of the second heart sound were critical ‘red flags’ that pointed toward a vascular aetiology. A contrast-enhanced CT scan revealed:

- Dilation of the aortic arch and descending thoracic aorta (6 cm diameter), Figs. 1, 2 and 3.
- Dissection extending from the arch (after the left subclavian artery) to the abdominal aorta with mural thrombi, causing marked luminal narrowing of the involved vessels. Figure 4a and d, and 5.
- Mural thrombi causing luminal narrowing at the origins of the renal and splanchnic vessels, leading to hypoperfusion. Figures 6A and D and 7.
- Serum Cr − 83µmol/L; WBC – 7.6 × 10^9^/L.

**Fig. 5:**
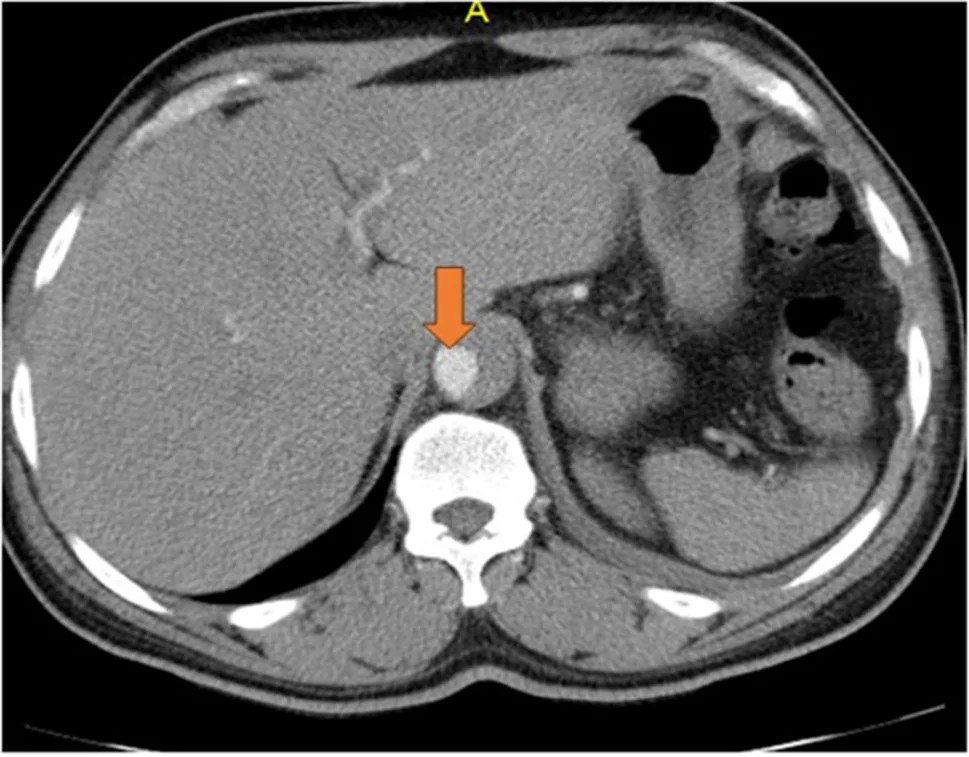
Post-contrast image showing the distal extension of the dissection into the abdominal aorta, with marked luminal narrowing of the true lumen (orange) due to the expansion of the false lumen

**Fig. 6:**
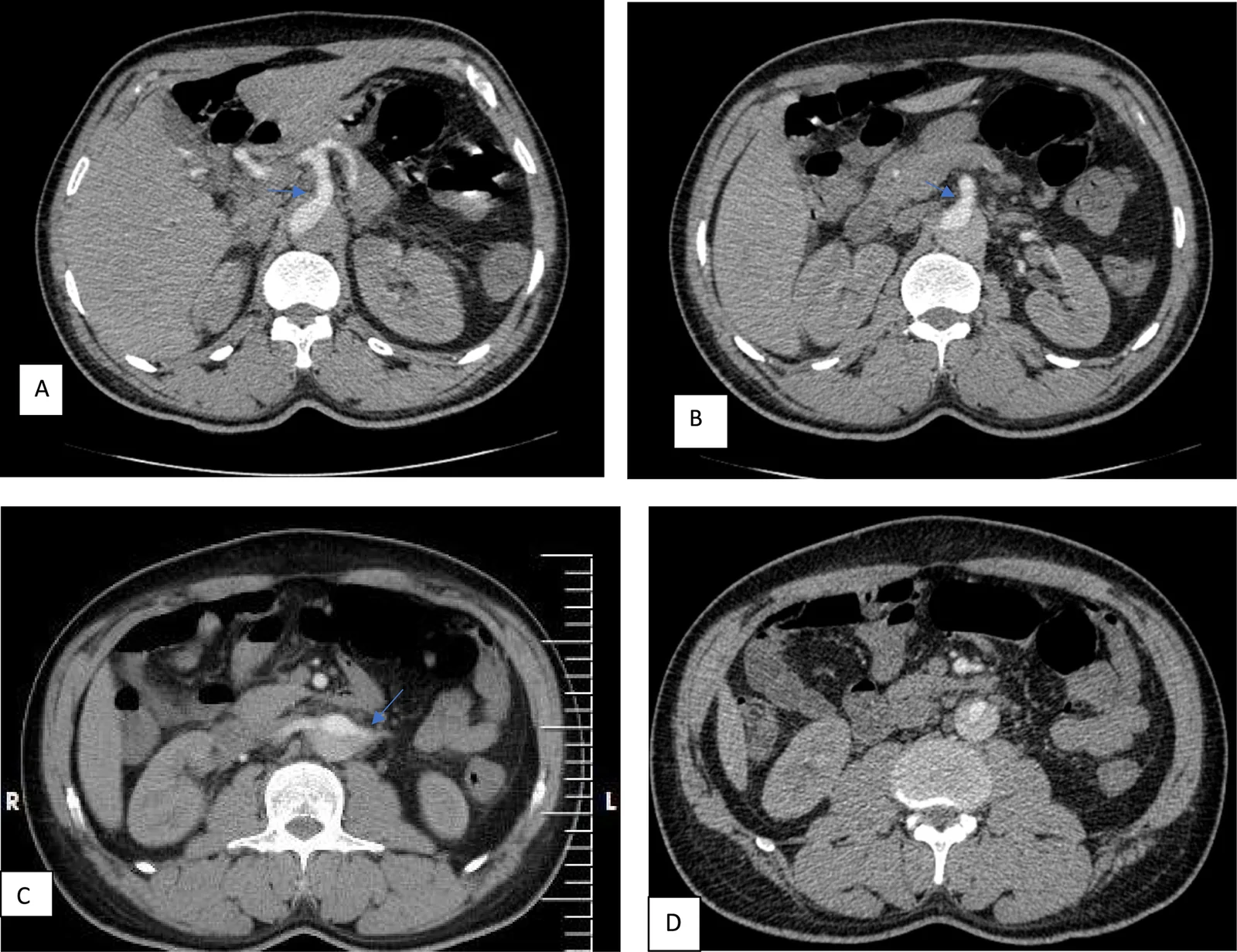
**A-D**: Serial Post-contrast axial CT (upper abdomen slices) showing the distal extent of the dissection with progressive narrowing of the abdominal aorta. **A** shows the origin of the coeliac artery (arrow); **B**, the Superior mesenteric artery (arrow); **C**, the renal arteries with mural thrombus within the false lumen compressing the true lumen, resulting in narrowing of the left renal artery (arrow); **D** is the most distal cut of the infrarenal aorta demonstrated

**Fig. 7:**
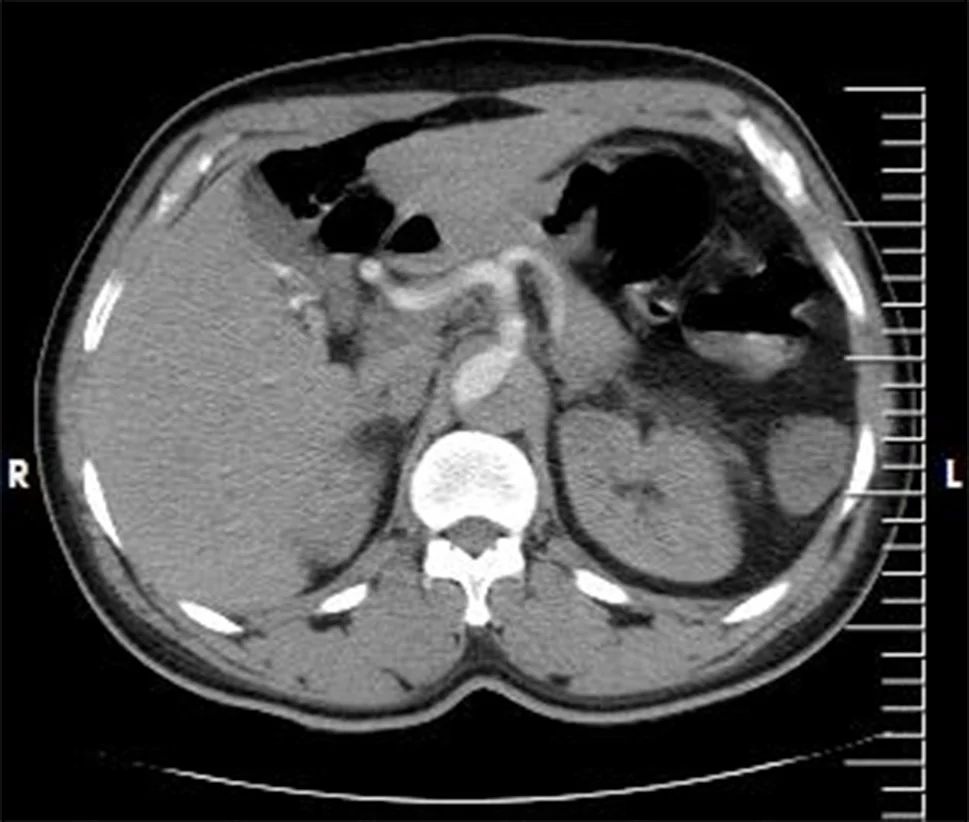
Reconstructed lower slice CT showing thrombi at the origin of the splanchnic (mesenteric) vessels which suggest the mechanism for mesenteric hypoperfusion, which clinically manifested as symptoms of bowel obstruction

### Therapeutic intervention

The patient was initially managed in the accident and emergency section. He was subsequently transferred to the surgical ward for ongoing care. The patient was stabilized by an interdisciplinary team (comprising Surgery, Cardiology, and Radiology subspecialties) for immediate vascular management, thereby avoiding an unnecessary and potentially catastrophic exploratory laparotomy. The established clinical targets for anti-impulse therapy were a systolic blood pressure below 140 mmHg, a diastolic blood pressure below 80 mmHg, and a resting heart rate at or below 80 beats per minute (bpm).

Prior to the initiation of intravenous (IV) therapy, the patient’s hemodynamic parameters were highly unstable; his blood pressure fluctuated between 180 and 200 mmHg systolic and 130–140 mmHg diastolic, accompanied by a tachycardic pulse rate of 120 bpm. IV labetalol was administered at a dose of 25 mg over 5 min to acutely lower both systemic vascular resistance and cardiac contractility, supplemented subsequently by oral antihypertensive medications (alpha methyl DOPA at 500 mg tds and lisinopril at 10 mg OD). Following this pharmacological regimen, the patient’s vitals moved steadily toward target goals, stabilizing at a blood pressure of 140/90 mmHg and a pulse rate of 84 bpm. Intravenous tramadol was utilized concurrently for effective pain titration.

### Follow-up and outcomes

Due to the patient’s request for transfer to a location of his choice, he was referred accordingly on the 10th day of admission and eventually had successful cardiothoracic evaluation and treatment at John H. Strager Hospital of Cook County (Division of Cardiothoracic Surgery). Thereafter, follow-up treatment is no longer within our purview.

## Discussion

### Atypical presentation and the “great masquerader”

Acute aortic dissection (AAD) is often termed “the great masquerader” due to its ability to mimic more common conditions [2]. While the classic presentation involves sudden, “tearing” chest or back pain, studies from the International Registry of Acute Aortic Dissection (IRAD) show that approximately 5% to 15% of cases involve gastrointestinal symptoms [3]. Pain can even be absent in up to 40% of mesenteric ischemia cases associated with AD, further complicating the diagnosis [8, 9]. This patient’s presentation - solely focused on constipation, distension, and colicky pain - is highly unusual and typically leads to an initial diagnosis of organic bowel obstruction, as seen here [10, 11].

Aortic anatomy and classification The CT Imaging of this case revealed that Fig. 4c and d demonstrate the communication (entry tear) between the true and false lumens in the descending thoracic aorta. This is the proximal site where the intimal disruption allows blood to enter the false lumen. The dissection originates from the aortic arch passed the left subclavian artery (proximal extent of the dissection), as seen in Fig. 4a and b, where an intimal flap is visible separating the true and false lumens at the arch level. There is significant aneurysmal dilation of the aortic arch (approximately 6 cm diameter, Fig. 1) and descending thoracic aorta (Fig. 2). The images do not show involvement of the left subclavian artery, suggesting that the dissection spare this branch or its involvement is minimal. The dissection involves primarily the aortic arch beyond the subclavian artery origin and descending thoracic aorta. The true lumen is compressed and narrowed due to the false lumen expansion, which contains mural thrombi (Figs. 4a and 6a and d). This compression compromises downstream perfusion, particularly to vital abdominal organs.

Based on the location and extent, this dissection falls under Stanford Type B, as it originates after the left subclavian artery extending to the descending thoracic and abdominal aorta without ascending aorta involvement. This corresponds to DeBakey Type IIIb [12].

The imaging findings indicate an aneurysmal dilation of the aorta measuring approximately 6 cm in diameter (Figs. 1, 2 and 3), which exceeds the normal upper limit for the thoracic aorta (typically < 4 cm). Such significant dilation suggests pre-existing aortic pathology, likely a chronic aneurysmal process, which may have predisposed the patient to the acute dissection event. This interpretation is further supported by the presence of mural thrombi within the false lumen (Fig. 4a and d) which may indicate chronicity, as organized thrombus formation often develops over time in chronic dissections. The patient’s longstanding hypertension (20 years) is a well-known risk factor for progressive aortic wall degeneration and aneurysm formation, setting the stage for an acute dissection superimposed on chronic disease [12].

### Pathophysiology of mesenteric malperfusion

In this case, the Stanford Type B dissection extended into the abdominal aorta, causing mesenteric malperfusion [10]. This occurs via two primary mechanisms: dynamic obstruction, where the dissection flap intermittently covers branch vessel origins, and static obstruction, where the flap or a thrombus permanently occludes the vessel [9]. The resulting hypoperfusion can lead to paralytic ileus or bowel wall ischemia, which clinically mimics a large bowel obstruction and explains the “distended bowel” seen on this patient’s initial X-rays.

### Abdominal presentations and management

The patient’s presentation with constipation, abdominal distension, and colicky pain initially suggested a large bowel obstruction. However, the CT imaging and clinical context clarify that this was not a mechanical bowel obstruction. No evidence of a mechanical transition point was described on CT, which is a hallmark of true mechanical obstruction. There was no report of any localized obstructing lesion or discrete transition zone on CT.

The distal extent of the dissection could not be determined beyond the image provided by the chest CT on this case because of resource limitation. This is a limitation of this study. Ideally, the aorta should have been imaged beyond the dissection via abdominal CT. The final acquired image was at the infrarenal abdominal aorta, where the false lumen remained larger than the true lumen with worsening of the luminal narrowing, raising the possibility of distal extension beyond the field of view, including potential involvement of the inferior mesenteric artery (IMA). The plain radiograph from the referral centre reported dilatation of the descending colon which is in the IMA territory.

The bowel findings are instead consistent with mesenteric hypoperfusion and ischemia caused by aortic dissection-related malperfusion. The hypoperfusion results in bowel wall ischemia and dysfunction, which clinically mimic obstruction through paralytic ileus or pseudo-obstruction (functional obstruction without a physical blockage). The CT findings noted mural thrombi compressing the true lumen at origins of renal and splanchnic vessels (Figs. 6A and D and 7), which supports compromised mesenteric blood flow leading to bowel dysmotility. No specific bowel wall thickening or ischemic changes were explicitly observed on CT, but the clinical picture and vascular compromise indicate mesenteric ischemia-induced paralytic ileus or pseudo-obstruction rather than mechanical obstruction. Thus, the patient’s bowel symptoms were secondary to functional impairment from mesenteric hypoperfusion caused by the aortic dissection, not from an intrinsic mechanical large bowel obstruction. This explains why surgical intervention for bowel obstruction was avoided and medical management for vascular stabilization was prioritized.

### Clinical red flags and diagnostic imaging

The diagnostic challenge was compounded by the patient’s history of constipation and colicky abdominal pain. However, persistent, severe hypertension (200/140 mmHg) and a “loud” aortic component of the second heart sound served as critical “red flags”. Literature emphasizes that hypertension is a factor in approximately 70% of AAD cases [8]. In patients with an acute surgical abdomen and severe hypertension, contrast-enhanced CT remains the gold standard for probing vascular integrity, as it provides high sensitivity and specificity for identifying the intimal flap and true/false lumens [10]. This patient’s aortic dissection qualifies as a complicated Stanford Type B dissection, as evidenced by imaging and clinical findings namely: severe compression of the true lumen by the expanding false lumen with mural thrombi (Figs. 4a and b and 5, and Fig. 6A and B), leading to significant luminal narrowing; hypoperfusion of branch vessels, specifically the renal and splanchnic (mesenteric) arteries, due to thrombus-induced obstruction at their origins (Figs. 6A and B and 7); and clinical manifestations consistent with branch vessel ischemia, such as bowel obstruction symptoms from mesenteric hypoperfusion [13, 14].

### Risks of inappropriate surgical intervention

Misdiagnosing AAD as a primary surgical abdomen can lead to inappropriate exploratory laparotomy, which carries a prohibitive mortality risk [10]. Studies indicate that patients with undiagnosed mesenteric malperfusion have mortality rates as high as 63% to 100% if not managed correctly [9]. Surgical intervention in the presence of an untreated aortic dissection can exacerbate instability, whereas stabilizing the patient with anti-impulse therapy (e.g., parenteral labetalol) to reduce blood pressure and cardiac contractility is the established medical priority for Stanford Type B dissections [1, 2, 9, 10].

### Pharmacological rationale: labetalol

The choice of parenteral labetalol for this patient was based on both clinical necessity and resource accessibility in the Nigerian healthcare setting. The patient was stabilized with intravenous labetalol, achieving controlled blood pressure and reduced aortic wall shear stress, which is the cornerstone of acute Type B dissection management [13, 14]. Absence of hemodynamic instability, clinical Improvement and controlled Symptoms allowed a non-emergent approach. Other rationale for medical management and transfer include: risk-benefit considerations and multidisciplinary consensus [13, 14]. In view of our limited resources to advanced endovascular or surgical facilities required for thoracic endovascular aortic repair or open surgery, patient was referred to appropriate centre of choice. Transfer ensured access to specialized vascular surgical expertise for further evaluation and possible intervention tailored to the patient’s evolving clinical status.

### Anti-impulse therapy

In Stanford Type B dissection, the primary goal is anti-impulse therapy [15]. By decreasing the heart rate (negative chronotropy) and the rate of left ventricular pressure rise, labetalol reduces the shear stress on the aortic wall [10]. This prevents the further propagation of the dissection flap and minimizes the risk of aortic rupture [15].

### Safety and monitoring

In this case, labetalol allowed for rapid stabilization of a critical blood pressure of 200/140 mmHg. Its predictable onset and duration made it a safer choice than pure vasodilators like hydralazine, which can inadvertently increase and worsen the dissection [1, 6, 7].

## Conclusions (The key “take-away” lessons are)

1. AD should be considered in hypertensive patients with abdominal symptoms.
2. Although the dissection is acute, the aneurysmal dilation and thrombus suggest an underlying chronic remodeling process.
3. Severe high blood pressure and atypical pain patterns are critical indicators of vascular etiology.
4. Imaging to probe the vascular integrity of the aorta is essential before surgical intervention in this setting.
5. Prioritizing advanced imaging should be the rule if a vascular cause is suspected. The clinicians should bypass serial abdominal films and proceed directly to contrast-enhanced CT (CTA). Relying on X-rays alone may lead to an inappropriate surgical referral and a potentially fatal exploratory laparotomy.

## Supplementary Information

Below is the link to the electronic supplementary material.

Supplementary Material 1

## Data Availability

No datasets were generated or analysed during the current study.

## Declarations

### Patient perspective

Patient was initially concerned because he thought he was simply suffering from a severe case of constipation. But the pain in his abdomen and back became almost unbearable. He was grateful that the doctors looked beyond his stomach issues to find the true cause of the pain, preventing an unnecessary surgery.

## Abbreviations

AAD: Acute Aortic Dissection
AD: Aortic Dissection
AED: Automated External Defibrillator
BP: Blood Pressure
BPH: Benign Prostatic Hypertrophy
bpm: Beats per minute
CT: Computed Tomography
CTA: Computed Tomography Angiography
FETHI: Federal Teaching Hospital Ido-Ekiti
GI: Gastrointestinal
IRAD: International Registry of Acute Aortic Dissection
IV: Intravenous
