# Supplementary material for: Acute aortic dissection masquerading as large bowel obstruction: a case report
Source: Egypt Heart J. 2026 Sep 17;78:68. doi: 10.1186/s43044-026-00775-y (PMC13586086; doi:10.1186/s43044-026-00775-y)
Supplement: Supplementary file 1 [file 43044_2026_775_MOESM1_ESM.docx]

**CARE Checklist**

Below is the **CARE Checklist** for the manuscript, indicating the specific location of each required element in the manuscript.

| **Item** | **Checklist Item** | **Manuscript Status / Location in the manuscript** |
| --- | --- | --- |
| **1** | **Title** | Present: "Acute Aortic Dissection Masquerading as Large Bowel Obstruction" (p. 1). |
| **2** | **Keywords** | Present: Aortic dissection, abdomen, surgery, hypertension, case report (p. 1). |
| **3a** | **Abstract: Background** | Present: AD is life-threatening and mimics acute surgical abdomen (p. 1). |
| **3b** | **Abstract: Case Presentation** | Present: 50-year-old male with constipation and distension (p. 1). |
| **3c** | **Abstract: Conclusions** | Present: Underscores the need for high suspicion of AD in hypertensive patients (p. 1). |
| **4** | **Introduction** | Present: Discusses AD and typical vs. atypical symptoms (p. 2). |
| **5a** | **Patient Information** | Present: 50-year-old Nigerian male (p. 2). |
| **5b** | **Medical History** | Present: 20-year hypertension, BPH; non-smoker, non-diabetic (p. 2). |
| **5c** | **Relevant Characteristics** | **Not Applicable/Not Mentioned**: No genetic or family history detailed in the PDF. |
| **6** | **Clinical Findings** | Present: Distress, tachycardia (120 bpm), BP (180/130 to 200/140), abdominal signs (p. 2). |
| **7** | **Timeline** | Present: Detailed table covering Day 1 through Day 8 (p. 3). |
| **8a** | **Diagnostic Assessment** | Present: Initial diagnosis (obstruction) vs. final diagnosis (Type B AD) (p. 3). |
| **8b** | **Diagnostic Challenges** | Present: Atypical GI symptoms and non-specific X-ray findings mentioned in the discussion (p. 9). |
| **8c** | **Diagnostic Reasoning** | Present: Use of contrast-enhanced CT to probe vascular integrity (pp. 3, 10). |
| **8d** | **Prognostic Characteristics** | **Not Applicable**: Formal prognostic scoring was not included. |
| **9a** | **Therapeutic Intervention** | Present: Conservative management with IV labetalol and IV tramadol (p. 8). |
| **9b** | **Administration Rationale** | Present: Rationale for anti-impulse therapy and dual blockade is mentioned (pp. 10-11). |
| **10a** | **Follow-up/Outcomes** | Present: Stabilization and referral/transfer to another facility (p. 8). |
| **10b** | **Important Test Results** | **Not Applicable**: Post-intervention follow-up imaging was not included in this report. |
| **10c** | **Intervention Adherence** | **Not Applicable**: Hospital-administered parenteral therapy. |
| **10d** | **Adverse/Unintended Events** | **Not Applicable**: No adverse reactions were explicitly reported in the PDF. |
| **11a** | **Discussion: Strengths** | Present: Avoiding unnecessary laparotomy is framed as a strength (p. 10). |
| **11b** | **Discussion: Limitations** | **Not Applicable**: Limitations of this specific case study were not explicitly discussed. |
| **11c** | **Relevant Literature** | Present: Mentions data from the International Registry of Acute Aortic Dissection (IRAD) (p. 9). |
| **11d** | **Rationale for Conclusions** | Present: Summarized in the "take-away lessons" (p. 11). |
| **12** | **Patient Perspective** | Present: paraphrase from the patient (p. 12). |
| **13** | **Informed Consent** | Present: Written informed consent obtained for publication (p. 12). |
